# 5-Chloro­pyrimidin-2-amine

**DOI:** 10.1107/S1600536809043645

**Published:** 2009-10-28

**Authors:** Bin Wang, Ran-Zhe Lu, Lu-Na Han, Wen-Bin Wei, Hai-Bo Wang

**Affiliations:** a College of Food Science and Light Industry, Nanjing University of Technology, Xinmofan Road No. 5 Nanjing, Nanjing 210009, People’s Republic of China; b College of Science, Nanjing University of Technology, Xinmofan Road No. 5 Nanjing, Nanjing 210009, People’s Republic of China

## Abstract

The complete mol­ecule of the title compound, C_4_H_3_ClN_3_, is generated by crystallographic mirror symmetry, with the Cl atom, one N atom and two C atoms lying on the reflecting plane. In the crystal structure, inter­molecular N—H⋯N hydrogen bonds link the mol­ecules into chains propagating in [100].

## Related literature

For general background, see: Hannouta & Johnson (1982 ▶). For bond-length data, see: Allen *et al.* (1987 ▶).
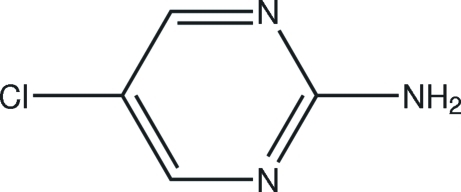


## Experimental

### 

#### Crystal data

C_4_H_4_ClN_3_
*M* *_r_* = 129.55
Orthorhombic, 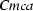

*a* = 7.6380 (15) Å
*b* = 8.2240 (16) Å
*c* = 17.100 (3) Å
*V* = 1074.1 (4) Å^3^
*Z* = 8
Mo *K*α radiation
μ = 0.59 mm^−1^
*T* = 293 K
0.30 × 0.20 × 0.10 mm

#### Data collection

Enraf–Nonius CAD-4 diffractometer
Absorption correction: ψ scan (North *et al.*, 1968 ▶) *T*_min_ = 0.844, *T*_max_ = 0.944
1047 measured reflections
534 independent reflections
462 reflections with *I* > 2˘*I*)
*R*_int_ = 0.020
3 standard reflections every 200 reflections intensity decay: 1%

#### Refinement

*R*[*F* ^2^ > 2σ(*F* ^2^)] = 0.033
*wR*(*F* ^2^) = 0.116
*S* = 0.92
534 reflections
44 parameters
H-atom parameters constrained
Δρ_max_ = 0.20 e Å^−3^
Δρ_min_ = −0.22 e Å^−3^

### 

Data collection: *CAD-4 EXPRESS* (Enraf–Nonius, 1994 ▶); cell refinement: *CAD-4 EXPRESS*; data reduction: *XCAD4* (Harms & Wocadlo, 1995 ▶); program(s) used to solve structure: *SHELXS97* (Sheldrick, 2008 ▶); program(s) used to refine structure: *SHELXL97* (Sheldrick, 2008 ▶); molecular graphics: *SHELXTL* (Sheldrick, 2008 ▶); software used to prepare material for publication: *PLATON* (Spek, 2009 ▶).

## Supplementary Material

Crystal structure: contains datablocks global, I. DOI: 10.1107/S1600536809043645/hb5158sup1.cif

Structure factors: contains datablocks I. DOI: 10.1107/S1600536809043645/hb5158Isup2.hkl

Additional supplementary materials:  crystallographic information; 3D view; checkCIF report

## Figures and Tables

**Table 1:** Hydrogen-bond geometry (Å, °)

*D*—H⋯*A*	*D*—H	H⋯*A*	*D*⋯*A*	*D*—H⋯*A*
N2—H2*A*⋯N1^i^	0.90	2.22	3.087 (2)	161

Symmetry code: (i) 
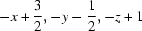
.

**Fig. 1.**
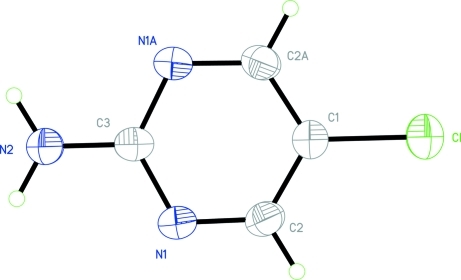
The molecular structure of (I) showing 50% displacement ellipsoids.

## supplementary crystallographic information

### Experimental

Guanidine (20 g) and 2-chloromalonaldehyde (16 g) were added to concentrated H_2_SO_4_ (50g) with cooling; the mixture was allowed to stand for two hours at room temperature, the product poured into ice water, neutralized with NH_4_OH, the precipitated filtered, made strongly alkaline with NH_4_OH, and the precipitate was recrystallized from alcohol or sublimed to give the title compound. Colourless blocks of (I) were obtained by slow evaporation of an methanol solution.

### Refinement

H atoms were positioned geometrically (N—H = 0.86 Å, C—H = 0.93–0.98Å) and refined as riding with U_iso_(H) = 1.2U_eq_(C,N) or 1.5U_eq_(methyl C).

### Crystal data

**Table:**

C_4_H_4_ClN_3_	*D*_x_ = 1.602 Mg m^−^^3^
*M**_r_* = 129.55	Melting point: 506 K
Orthorhombic, *C**m**c**a*	Mo *K*α radiation, λ = 0.71073 Å
Hall symbol: -C 2bc 2	Cell parameters from 25 reflections
*a* = 7.6380 (15) Å	θ = 10–13°
*b* = 8.2240 (16) Å	µ = 0.59 mm^−^^1^
*c* = 17.100 (3) Å	*T* = 293 K
*V* = 1074.1 (4) Å^3^	Block, colourless
*Z* = 8	0.30 × 0.20 × 0.10 mm
*F*(000) = 528	

### Data collection

**Table:**

Enraf–Nonius CAD-4 diffractometer	462 reflections with *I* > 2˘*I*)
Radiation source: fine-focus sealed tube	*R*_int_ = 0.020
graphite	θ_max_ = 25.3°, θ_min_ = 2.4°
ω/2θ scans	*h* = 0→9
Absorption correction: ψ scan (North *et al.*, 1968)	*k* = 0→9
*T*_min_ = 0.844, *T*_max_ = 0.944	*l* = −20→20
1047 measured reflections	3 standard reflections every 200 reflections
534 independent reflections	intensity decay: 1%

### Refinement

**Table:**

Refinement on *F*^2^	Secondary atom site location: difference Fourier map
Least-squares matrix: full	Hydrogen site location: inferred from neighbouring sites
*R*[*F*^2^ > 2σ(*F*^2^)] = 0.033	H-atom parameters constrained
*wR*(*F*^2^) = 0.116	*w* = 1/[σ^2^(*F*_o_^2^) + (0.1*P*)^2^ + 0.2*P*] where *P* = (*F*_o_^2^ + 2*F*_c_^2^)/3
*S* = 0.92	(Δ/σ)_max_ < 0.001
534 reflections	Δρ_max_ = 0.20 e Å^−^^3^
44 parameters	Δρ_min_ = −0.22 e Å^−^^3^
0 restraints	Extinction correction: *SHELXL97* (Sheldrick, 2008), Fc^*^=kFc[1+0.001xFc^2^λ^3^/sin(2θ)]^-1/4^
Primary atom site location: structure-invariant direct methods	Extinction coefficient: 0.025 (4)

### Special details

**Table:**

Geometry. All esds (except the esd in the dihedral angle between two l.s. planes) are estimated using the full covariance matrix. The cell esds are taken into account individually in the estimation of esds in distances, angles and torsion angles; correlations between esds in cell parameters are only used when they are defined by crystal symmetry. An approximate (isotropic) treatment of cell esds is used for estimating esds involving l.s. planes.
Refinement. Refinement of F^2^ against ALL reflections. The weighted R-factor wR and goodness of fit S are based on F^2^, conventional R-factors R are based on F, with F set to zero for negative F^2^. The threshold expression of F^2^ > 2sigma(F^2^) is used only for calculating R-factors(gt) etc. and is not relevant to the choice of reflections for refinement. R-factors based on F^2^ are statistically about twice as large as those based on F, and R- factors based on ALL data will be even larger.

### Fractional atomic coordinates and isotropic or equivalent isotropic displacement parameters (Å^2^)

**Table:**

	*x*	*y*	*z*	*U*_iso_*/*U*_eq_	
Cl	0.5000	0.22728 (9)	0.27440 (4)	0.0527 (4)	
N1	0.65713 (16)	−0.11849 (18)	0.41923 (8)	0.0360 (5)	
C3	0.5000	−0.1761 (3)	0.44284 (14)	0.0319 (6)	
N2	0.5000	−0.3014 (3)	0.49308 (15)	0.0432 (7)	
H2A	0.6021	−0.3434	0.5099	0.052*	
C2	0.6542 (2)	0.0027 (2)	0.36821 (10)	0.0364 (5)	
H2C	0.7598	0.0457	0.3507	0.044*	
C1	0.5000	0.0674 (3)	0.34019 (13)	0.0347 (6)	

### Atomic displacement parameters (Å^2^)

**Table:**

	*U*^11^	*U*^22^	*U*^33^	*U*^12^	*U*^13^	*U*^23^
Cl	0.0488 (6)	0.0514 (6)	0.0580 (6)	0.000	0.000	0.0225 (3)
N1	0.0258 (9)	0.0398 (9)	0.0424 (9)	−0.0007 (6)	0.0004 (5)	0.0045 (6)
C3	0.0278 (12)	0.0325 (13)	0.0354 (12)	0.000	0.000	−0.0039 (11)
N2	0.0288 (11)	0.0450 (13)	0.0559 (14)	0.000	0.000	0.0179 (11)
C2	0.0296 (10)	0.0379 (10)	0.0418 (9)	−0.0033 (7)	0.0032 (7)	0.0013 (7)
C1	0.0353 (13)	0.0328 (13)	0.0359 (13)	0.000	0.000	0.0026 (10)

### Geometric parameters (Å, °)

**Table:**

Cl—C1	1.730 (2)	N2—H2A	0.9000
N1—C2	1.325 (2)	C2—C1	1.378 (2)
N1—C3	1.3519 (18)	C2—H2C	0.9300
C3—N2	1.341 (4)	C1—C2^i^	1.378 (2)
C3—N1^i^	1.3519 (18)		
			
C2—N1—C3	116.44 (14)	N1—C2—H2C	118.9
N2—C3—N1^i^	117.41 (11)	C1—C2—H2C	118.9
N2—C3—N1	117.41 (11)	C2—C1—C2^i^	117.4 (2)
N1^i^—C3—N1	125.2 (2)	C2—C1—Cl	121.28 (11)
C3—N2—H2A	120.0	C2^i^—C1—Cl	121.28 (11)
N1—C2—C1	122.25 (15)		
			
C2—N1—C3—N2	178.5 (2)	N1—C2—C1—C2^i^	1.0 (4)
C2—N1—C3—N1^i^	−0.8 (4)	N1—C2—C1—Cl	179.28 (14)
C3—N1—C2—C1	−0.1 (3)		

Symmetry codes: (i) −*x*+1, *y*, *z*.

### Hydrogen-bond geometry (Å, °)

**Table:**

*D*—H···*A*	*D*—H	H···*A*	*D*···*A*	*D*—H···*A*
N2—H2A···N1^ii^	0.90	2.22	3.087 (2)	161

Symmetry codes: (ii) −*x*+3/2, −*y*−1/2, −*z*+1.

## References

[bb1] Allen, F. H., Kennard, O., Watson, D. G., Brammer, L., Orpen, A. G. & Taylor, R. (1987). *J. Chem. Soc. Perkin Trans. 2*, pp. S1–19.

[bb2] Enraf–Nonius (1994). *CAD-4 EXPRESS* Enraf–Nonius, Delft, The Netherlands.

[bb4] Hannouta, I. B. & Johnson, A. (1982). *Dyes Pigments*, **3**, 173–182.

[bb5] Harms, K. & Wocadlo, S. (1995). *XCAD4* University of Marburg, Germany.

[bb6] North, A. C. T., Phillips, D. C. & Mathews, F. S. (1968). *Acta Cryst.* A**24**, 351–359.

[bb7] Sheldrick, G. M. (2008). *Acta Cryst.* A**64**, 112–122. 10.1107/S0108767307043930 18156677

[bb8] Spek, A. L. (2009). *Acta Cryst.* D**65**, 148–155. 10.1107/S090744490804362X PMC2631630 19171970

